# Cardiomyopathy Screening Adherence Among Medicaid‐Enrolled Long‐Term Survivors of Childhood Cancer

**DOI:** 10.1002/cam4.71116

**Published:** 2025-08-05

**Authors:** Xin Hu, Sharon M. Castellino, Deo Kumar Srivastava, Paul C. Nathan, Gregory T. Armstrong, Claire Snyder, Anne C. Kirchhoff, Xu Ji

**Affiliations:** ^1^ Department of Radiation Oncology Emory University School of Medicine Atlanta Georgia USA; ^2^ Department of Pediatrics Emory University School of Medicine Atlanta Georgia USA; ^3^ Winship Cancer Institute Emory University Atlanta Georgia USA; ^4^ Aflac Cancer & Blood Disorders Center Children's Healthcare of Atlanta Atlanta Georgia USA; ^5^ Biostatistics Department St. Jude Children's Research Hospital Memphis Tennessee USA; ^6^ The Hospital for Sick Children and University of Toronto Toronto Ontario Canada; ^7^ Department of Epidemiology and Cancer Control St. Jude Children's Research Hospital Memphis Tennessee USA; ^8^ Department of Medicine Johns Hopkins University School of Medicine Baltimore Maryland USA; ^9^ Department of Pediatrics University of Utah School of Medicine Salt Lake City Utah USA; ^10^ Cancer Control and Population Sciences Huntsman Cancer Institute Salt Lake City Utah USA

## Abstract

**Background:**

Childhood cancer survivors face increased risks of adverse cardiovascular outcomes due to treatment exposures. National guidelines recommend periodic cardiomyopathy screening for survivors exposed to anthracyclines or radiation. We examined the receipt of and the adherence to guideline‐recommended cardiomyopathy testing (echocardiogram, multigated acquisition scan, magnetic resonance imaging) among Medicaid‐enrolled childhood cancer survivors.

**Method:**

Using data from the Childhood Cancer Survivor Study linked to administrative Medicaid claims, we analyzed 1062 at‐risk survivors aged 18–64 years who were continuously enrolled in Medicaid each year throughout 2015–2019.

**Results:**

We found that 26.8% received any cardiomyopathy test, and 9.6% adhered to guidelines. Residence in Medicaid expansion (vs. non‐expansion) states was associated with 6.9% points (95% CI = 0.7–13.1) higher likelihood of receiving any cardiomyopathy test and 4.2% points (95% CI = 0.4–7.9) higher likelihood of adherence.

**Conclusions:**

These findings suggest low receipt and poor adherence to guideline‐recommended cardiomyopathy screening among long‐term survivors of childhood cancer. Our data further underscore the critical role of Medicaid programs in supporting access to quality survivorship care for this vulnerable population.

Childhood cancer survivors face increased risks of adverse cardiovascular outcomes, such as heart failure and myocardial dysfunction, due to cancer treatment exposure [1, 2, 3]. The Children's Oncology Group (COG) and the International Guideline Harmonization Group (IGHG) recommend cardiomyopathy screening—specifically echocardiograms or equivalent imaging—for survivors exposed to anthracyclines and/or radiation [4]. However, evidence reveals gaps in screening receipt. For example, roughly 40% of privately insured anthracycline‐treated survivors in the U.S. did not receive any cardiomyopathy testing within 5 years post‐treatment [5]. Importantly, the patterns of receipt and adherence to guideline‐recommended cardiomyopathy screening among Medicaid‐enrolled survivors—a low‐income population disproportionately subject to barriers to healthcare—remain poorly understood.

Access to quality health insurance is strongly linked to timely, appropriate survivorship care [6]. Medicaid expansion under the Affordable Care Act aimed to increase insurance coverage and healthcare access for low‐income adults. By the end of 2019, Medicaid expansion had been implemented in 34 states [7], with mounting evidence of its role in improving cancer diagnosis, treatment initiation, and survival outcomes [8, 9, 10, 11]. However, whether Medicaid expansion affects the receipt of cardiomyopathy screening and adherence to guidelines in long‐term childhood cancer survivors remains unknown.

We leveraged a unique linkage between the Childhood Cancer Survivor Study (CCSS) and Medicaid administrative claims to describe the patterns of cardiomyopathy screening and adherence to screening guidelines among Medicaid‐enrolled childhood cancer survivors. Additionally, we examined whether these patterns varied based on Medicaid expansion status in survivors' state of residence.

The CCSS cohort comprises 5‐year survivors diagnosed with cancer before age 21 years between 1970 and 1999 and treated at one of 31 participating institutions in North America, with detailed treatment exposures characterized [12]. For this analysis, we included CCSS survivors aged 18–64 years, residing in the U.S., alive, and continuously covered by Medicaid (defined as ≥ 6 months of enrollment each year) throughout our 5‐year study period in 2015–2019. A sensitivity analysis was conducted by requiring ≥ 11 months of Medicaid enrollment each year.

The cohort was restricted to “at‐risk” survivors, defined as those who received anthracycline chemotherapy, radiation to chest or heart, total body irradiation, or a combination of these treatments. To ensure the cardiomyopathy tests identified reflected screening rather than diagnostic or therapeutic purposes, we excluded survivors with a self‐reported history of grade 3–4 (severe or life‐threatening) cardiovascular conditions by 2019 [13]. We also excluded participants who moved between states with different Medicaid expansion statuses during the study period. Sample derivation is detailed in Figure S1.

Outcomes included (1) receipt of any cardiomyopathy testing (≥ 1 echocardiogram, multigated acquisition [MUGA] scan, and/or magnetic resonance imaging [MRI]) [5]; and (2) adherence to COG Guideline V4.0 recommended age‐ and therapy dose‐specific frequency of cardiomyopathy testing (hereafter “adherence”) [4]. Adherence was defined according to COG Guideline V4.0 to aligned with our study period. The Guideline recommended cardiomyopathy testing frequencies ranging from annually (e.g., receiving doxorubicin equivalent cumulative anthracycline dose of ≥ 200 mg/m^2^ before age 1 year) to every 5 years (e.g., receiving < 200 mg/m^2^ anthracyclines and no radiation at age 5 or older). See detailed adherence algorithm in Table S1.

We categorized Medicaid expansion status in survivors' resident states into three groups: expansion (27 states that implemented Medicaid expansion in 2014), late expansion (7 states that implemented Medicaid expansion between 2015 and 2019), and non‐expansion (17 states that had not implemented the expansion by the end of 2019; Table S2).

We constructed multivariable logistic regressions to assess the association between Medicaid expansion status and each outcome, adjusting for confounders selected based on Andersen's Behavioral Model of Health Services Use [14]. These included predisposing factors (age as of 2019, sex, race/ethnicity), enabling factors at both the individual level (Medicaid plan type, dual Medicare‐Medicaid eligibility, education attainment) and contextual level (ZIP Code‐level rurality and Distressed Communities Index [15]), and need factors (presence of grade 3–4 non‐cardiovascular medical conditions, presence of second cancer or recurrence). Standard errors were clustered at the ZIP Code level. Model‐adjusted probability differences (marginal effects [MEs]) and associated 95% confidence intervals (CIs) were reported [16].

Our sample included 1062 at‐risk survivors, with 57.0% female, 52.8% aged 27–39 years, and 72.8% non‐Hispanic White survivors (Table 1). Approximately one‐fourth (26.1%) received anthracycline only, 46.2% received radiation to the chest or heart or total body irradiation (no anthracycline), and 27.7% received both anthracycline and radiation therapy. Additionally, 57.4% of survivors resided in expansion states, while 17.4% resided in late‐expansion states and 25.1% in non‐expansion states.

**TABLE 1:** Factors associated with any cardiomyopathy screening and adherence to screening guidelines among at‐risk survivors.

Characteristics	*N*	Column %	Any cardiomyopathy test (yes vs. no)			Guideline adherence (yes vs. no)		
			Unadjusted % with any test (row %)	Adjusted probability difference (i.e., marginal effects)	*p*	Unadjusted% guideline adherent (row %)	Adjusted probability difference (i.e., marginal effects) (95% CI)	*p*
	1062	100.0%		(95% CI)				
**State policy‐level predictor**								
ACA Medicaid expansion status								
Non‐expansion states	267	25.1%	23.2%	Ref.		6.4%	Ref.	
2014 January expansion states	610	57.4%	28.5%	6.9 (0.7, 13.1)	0.03	10.2%	4.2 (0.4, 7.9)	0.03
Late expansion states	185	17.4%	26.5%	3.0 (−4.9, 10.9)	0.46	12.4%	6.3 (0.6, 11.9)	0.03
**ZIP Code‐level predictor**								
Rurality of residence^a^								
Small town or rural	488	46.0%	29.9%	Ref.		11.1%	Ref.	
Suburban	332	31.3%	24.4%	−4.7 (−11.3, 1.9)	0.16	7.5%	−3.9 (−8.2, 0.4)	0.08
Urban	242	22.8%	24.0%	−5.3 (−12.5, 1.8)	0.15	9.5%	−1.4 (−6.8, 3.4)	0.52
Neighborhood Distressed Communities Index^b^								
1 (prosperous)	204	19.2%	25.5%	Ref		9.8%	Ref.	
2	184	17.3%	23.4%	−0.8 (−9.4, 7.8)	0.86	8.2%	−2.4 (−8.6, 3.8)	0.44
3	202	19.0%	28.7%	4.1 (−4.7, 12.8)	0.36	10.9%	0.1 (−6.3, 6.4)	0.98
4	234	22.0%	27.8%	3.4 (−5.2, 12.0)	0.43	11.1%	0.2 (−6.5, 6.9)	0.95
5 (distressed)	238	22.4%	28.2%	4.0 (−5.2, 13.3)	0.39	8.0%	−3.4 (−9.9, 3.2)	0.31
**Individual‐level predictor**								
Age as of 2019 (years)								
27–39^c^	561	52.8%	25.3%	−1.4 (−6.9, 4.2)	0.63	8.2%	−3.1 (−6.7, 0.6)	0.10
40–63.8	501	47.2%	28.5%	Ref.		11.2%	Ref.	
Sex								
Male	457	43.0%	26.0%	Ref.		11.4%	Ref.	
Female	605	57.0%	27.4%	0.8 (−4.5, 6.0)	0.77	8.3%	−3.4 (−7.0, 0.3)	0.07
Race and ethnicity								
Non‐Hispanic White	773	72.8%	27.8%	Ref.		9.8%	Ref.	
Non‐Hispanic Black	108	10.2%	30.6%	6.9 (−3.8, 17.5)	0.21	9.3%	2.7 (−5.2, 10.5)	0.51
Hispanic, other, or missing	181	17.0%	20.4%	−4.5 (−11.9, 2.9)	0.23	8.8%	1.0 (−4.4, 6.5)	0.71
Medicaid health plan type^d^								
Fee for service	306	28.8%	30.1%	Ref.		10.5%	Ref.	
Comprehensive managed care	590	55.6%	24.6%	−3.9 (−10.6, 2.9)	0.26	8.3%	−1.2 (−5.7, 3.3)	0.60
Prepaid health plans	128	12.1%	26.6%	−1.9 (−11.2, 7.5)	0.69	10.9%	1.9 (−4.9, 8.6)	0.59
Behavioral health organization	38	3.6%	36.8%	7.2 (−10.9, 25.3)	0.44	18.4%	7.2 (−5.4, 19.8)	0.26
Dual Medicare‐Medicaid enrollment^e^								
No dual enrollment	552	52.0%	24.6%	Ref.		8.3%	Ref.	
Any month of dual enrollment	510	48.0%	29.2%	1.8 (−4.2, 7.7)	0.57	11.0%	1.0 (−3.0, 5.1)	0.62
Education attainment^f^								
Some college or equivalent, college graduate, or postgraduate	484	45.6%	27.9%	Ref.		8.9%	Ref.	
High school or lower, or unknown education status	578	54.4%	26.0%	−1.7 (−7.4, 4.0)	0.56	10.2%	1.7 (−2.1, 5.5)	0.37
Presence of grade 3–4 non‐cardiovascular medical conditions before 2015								
No	549	51.7%	24.4%	Ref.		8.2%	Ref.	
Yes	513	48.3%	29.4%	3.8 (−1.5, 9.2)	0.16	11.1%	2.8 (−0.9, 6.5)	0.14
Presence of second cancer or recurrence of primary malignancy before 2019								
No	860	81.0%	25.2%	Ref.		9.1%	Ref.	
Yes	202	19.0%	33.7%	6.4 (−0.7, 13.5)	0.08	11.9%	1.2 (−3.4, 5.9)	0.60

*Note:* At‐risk: those who received anthracycline chemotherapy, radiation (to chest or heart, total body irradiation), or a combination of these treatments.

Abbreviations: CI, confidence interval; Ref., reference.

^a^ This measure was based on the 2010 U.S. Census urban and rural classification and urban area criteria to classify a ZIP code as urban, suburban, small town, or rural.

^b^ Distressed Communities Index (DCI) was calculated based on seven neighborhood‐level measures: no high school diploma, housing vacancy rates, adults not working, poverty rate, median income ratio, changes in employment, and changes in establishments. The DCI was then classified into quintiles: prosperous (quintile 1), comfortable, mid‐tier, at risk, and distressed (quintile 5). The DCI used in this analysis was built from the U.S. Census Bureau's American Community Survey 5‐year estimates covering 2014–2018 and the Census Bureau's Business Patterns datasets for the same years.

^c^ The minimum age (as of 2019) of our analytic sample was 27 years old.

^d^ Medicaid plan type was measured in 2019.

^e^ Dual Medicare‐Medicaid enrollment was defined as at least 1 month enrolled in Medicare and Medicaid over the 5‐year study period (2015–2019).

^f^ The measure of education was from the most recent CCSS survey data within our study period; if this information was unavailable, we used data from the preceding survey that was most recent.

Between 2015 and 2019, 26.8% (*n* = 285) received any cardiomyopathy test; among test recipients, 94.4% underwent an echocardiogram, 22.8% a MUGA scan, and 1.4% an MRI. Overall, 9.6% (*n* = 102) survivors adhered to COG guideline‐recommended age‐ and therapy dose‐specific frequency of cardiomyopathy testing throughout 2015–2019 (Figure 1). The proportion of survivors receiving any cardiomyopathy testing was 28.5% in expansion states, 26.5% in late‐expansion states, and 23.2% in non‐expansion states (*p* = 0.26). Adherence rates were 10.2%, 12.4%, and 6.4% in expansion, late‐expansion, and non‐expansion states, respectively (*p* = 0.08).

**FIGURE 1:**
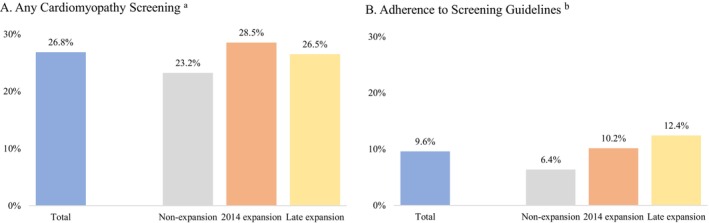
Unadjusted percentages of cancer survivors receiving any cardiomyopathy screening and adherent to screening guidelines.

In multivariable models, residence in expansion (vs. non‐expansion) states was associated with a 6.9 percentage points (ppts; 95% CI = 0.7–13.1, *p* = 0.03) higher likelihood of receiving any cardiomyopathy test, and a 4.2 ppts (95% CI = 0.4–7.9, *p* = 0.03) higher likelihood of adherence to guideline‐recommended testing frequencies (Table 1). Survivors in late‐expansion (versus non‐expansion) states showed a higher likelihood of receiving any cardiomyopathy test, despite statistically nonsignificant (ME = 3.0 ppts, 95% CI = −4.9–10.9, *p* = 0.46), and a 6.3 ppts (95% CI = 0.6–11.9, *p* = 0.03) higher likelihood of adherence. No other factors were statistically significantly associated with cardiomyopathy test receipt or adherence. The sensitivity analysis that restricting to those with ≥ 11 months of Medicaid enrollment each year throughout 2015–2019 showed consistent results (Figure S2, Table S3).

In this multi‐institutional cohort study, we provided the first evidence of the receipt and adherence to guideline‐concordant, risk‐based cardiomyopathy screening among Medicaid‐covered long‐term survivors of childhood cancer. Our results revealed low receipt; only 26.5% of at‐risk survivors received any cardiomyopathy testing over the five‐year study period. Adherence to guidelines was even lower, with approximately one‐in‐ten (9.6%) at‐risk survivors meeting guideline‐recommended screening frequencies. Notably, Medicaid expansion in survivors' state of residence was associated with notable increases in cardiomyopathy screening receipt and guideline adherence.

Our estimates of cardiomyopathy screening receipt differ from earlier studies, including that from our team showing a higher cardiomyopathy screening rate among privately insured early survivors, which may be due to variations in study design, data sources, patient populations, and assessment timeframes [5, 17]. In the current analysis, all survivors were ≥ 16 years from cancer diagnosis during our study period; this longer survivorship period may have resulted in reduced engagement with care teams and the broader healthcare system after transition to adult care, compared to those in earlier phases of survivorship [18]. Differences in screening receipt may also stem from differences in sociodemographic characteristics, insurance benefits, and healthcare access between Medicaid insured and privately insured populations [19].

The observed low screening rates may be attributable to several factors, including lack of patient awareness regarding their elevated health risks from cancer treatments, providers (particularly those outside of the oncology care team) unawareness of patients' risks, and limited familiarity with guidelines among adult oncology and non‐oncology providers [20, 21]. Even if some survivors may be aware of their screening needs, they may still face financial or logistical barriers to accessing screening [22]. Our findings underscore the urgent need for further research to identify barriers to screening among long‐term survivors, especially those insured through Medicaid, and to design targeted interventions that can engage both survivors and providers, promote adherence to screening guidelines, and ultimately improve cardiovascular health and survival in this high‐risk population.

Our finding of increased screening receipt and adherence in expansion states compared to non‐expansion states may reflect several unique features of Medicaid expansion. First, Medicaid expansion may have improved healthcare infrastructure within expansion states, such as by reducing uncompensated care [23, 24, 25] and improving the financial performance of hospitals and providers [24, 26], which may enhance the delivery of preventive care and adherence to clinical guidelines. Second, transportation services have been strengthened under Medicaid expansion [27], enabling enrollees without reliable transportation to attend medical appointments and reducing logistical barriers to accessing screening [28, 29]. Third, the expansion has increased enrollment in managed care plans, which often prioritize preventive care, patient outreach, and care coordination services [30], potentially helping survivors adhere to guideline‐based screening schedules.

This study has limitations. First, the cross‐sectional study design precludes causal inference between residency in Medicaid expansion states and screening utilization. Second, the analysis was restricted to survivors continuously enrolled in Medicaid during the study period, potentially limiting the generalizability of findings to survivors with other types of insurance or those uninsured. However, this cohort, linked to claims data, represents the best available data to examine “real‐world” adherence to screening guidelines among at‐risk long‐term childhood cancer survivors. Third, some survivors reporting grade 3–4 cardiovascular conditions during 2015–2019 may be diagnosed through screening; excluding them from analysis may have underestimated screening rates. Lastly, reliance on Medicaid claims data may have resulted in an underestimation of screening rates if some services were not billed through Medicaid, such as those paid for out‐of‐pocket. Nonetheless, this underestimation is likely minimal among our sample, which predominantly represents survivors with lower socioeconomic status and limited financial resources. Furthermore, any potential underestimation would occur consistently across states, minimizing its impact on the estimated differences between expansion and non‐expansion states.

In this retrospective study of Medicaid‐enrolled long‐term childhood cancer survivors, we observed low cardiomyopathy screening receipt and poor adherence to screening guidelines. Survivors in Medicaid expansion states had higher screening utilization and adherence, reflecting the beneficial role of the Medicaid programs in supporting care for socioeconomically disadvantaged survivors. These findings highlight the critical need for programs and policy reforms that can enhance access to guideline‐concordant care for childhood cancer survivors, a vulnerable population with many years of life ahead.

## Author Contributions

X.H.: conceptualized and designed the study, carried out the analyses, interpreted data, drafted the initial manuscript, and reviewed and revised the manuscript. S.M.C. and A.C.K.: conceptualized and designed the study, obtained funding, administered the project, interpreted data, and reviewed and revised the manuscript. D.K.S., P.C.N., G.T.A., and C.S.: conceptualized the study, interpreted data, and reviewed and revised the manuscript. X.J.: conceptualized and designed the study, obtained funding, administered the project, interpreted data, supervised project administration, and reviewed and revised the manuscript. All authors approved the final manuscript as submitted and agree to be accountable for all aspects of the work.

## Ethics Statement

This study was approved by the Institutional Review Boards of Emory University and St. Jude Children's Research Hospital, the CCSS coordinating center. Informed consent from the study populations is not required for this study.

## Conflicts of Interest

Authors received grants from the National Institutes of Health (S.M.C., X.J.), Emory University (X.J.), the Department of Defense (S.M.C.), Rally Foundation for Childhood Cancer Research (S.M.C., X.J.), the Leukemia & Lymphoma Society (S.M.C., X.J.), PhRMA Foundation (X.H.), St. Jude Children's Hospital (X.H.), and Pfizer (X.H.) outside the submitted work. S.M.C. serves on the Scientific Advisory Board (Pfizer [formerly Seagen Inc. and Brstol Meyers Squibb]). C.S. receives research funding from Pfizer through her institution and personal consulting fees from Shionogi (previous) and Movember (current). No other authors have conflicts of interest to disclose.

## Supporting information


**Data S1:** cam471116‐sup‐0001‐Supinfo.docx.

## Data Availability

The Childhood Cancer Survivor Study is a US National Cancer Institute funded resource (U24 CA055727) to promote and facilitate research among long‐term survivors of cancer diagnosed during childhood and adolescence. CCSS data are publicly available on dbGaP at https://www.ncbi.nlm.nih.gov/gap/ through its accession number phs001327.v2.p1. and on the St. Jude Survivorship Portal within the St. Jude Cloud at https://survivorship.stjude.cloud/. In addition, utilization of the CCSS data that leverages the expertise of CCSS Statistical and Survivorship research and resources will be considered on a case‐by case basis. For this utilization, a research Application of Intent followed by an Analysis Concept Proposal must be submitted for evaluation by the CCSS Publications Committee. Users interested in utilizing this resource are encouraged to visit http://ccss.stjude.org. Full analytical data sets associated with CCSS publications since January of 2023 are also available on the St. Jude Survivorship Portal at https://viz.stjude.cloud/community/cancer‐survivorship‐community~4/publications.
