# Supplementary material for: Cardiomyopathy Screening Adherence Among Medicaid‐Enrolled Long‐Term Survivors of Childhood Cancer
Source: Cancer Med. 2025 Aug 5;14(15):e71116. doi: 10.1002/cam4.71116 (PMC12322921; doi:10.1002/cam4.71116)
Supplement: Supplementary file 1 — Data S1: cam471116‐sup‐0001‐Supinfo.docx. [file CAM4-14-e71116-s001.docx]

**Online Supplement**

Figure S1. Sample Derivation Flowchart

Figure S2. Unadjusted Percentages of Cancer Survivors with ≥11 Months of Medicaid Enrollment Each Year Throughout 2015-2019 Who Received Any Cardiomyopathy Screening and Adhered to Screening Guidelines

Table S1. Algorithm Used to Define Adherence Based on the Children’s Oncology Group (COG) Guidelines, Version 4 ^a^

Table S2. States by Medicaid expansion status as of 2019

Table S3. Factors Associated With Any Cardiomyopathy Screening and Adherence to Screening Guidelines among At-Risk Survivors with ≥11 Months Medicaid Enrollment Each Year

Figure S1. Sample Derivation Flowchart

Exclude 7 participants with missing ZIP code information

**BOX 4:**

CCSS participants who resided in the U.S. and were aged 18-64 years throughout 2015-2019, alive by 2019, and with non-missing SSN (to be able to link to Medicaid data)

**N=13,917**

CCSS participants who enrolled in Medicaid in all years from 2015 to 2019

**N=3,348**

CCSS participants who had no self-reported Grade 3 to 4 cardiovascular conditions by 2019

**N=2,887**

Exclude 47 participants who moved between Medicaid expansion states and non-expansion states during the study period

CCSS participants who did not move across Medicaid expansion states and non-expansion states

**N=2,840**

CSSS participants continuously enrolled in Medicaid (defined as having at least six months of enrollment in each year) from 2015 to 2019

**N=1,449**

Exclude 10,569 participants not enrolled in Medicaid in 2015-2019

Exclude 1,771 participants who did not have continuous Medicaid enrollment during our study period

Exclude 461 participants who self-reported Grade 3 to 4 cardiovascular conditions by 2019

CSSS participants identified as at-risk survivors and without missing ZIP code

**N=1,062 (final analytic sample)**

Exclude 380 participants not identified as at-risk survivors (i.e., did not receive radiation to chest or heart, total body irradiation, or anthracyclines)

Notes: CCSS = Childhood Cancer Survivor Study; SSN=Social Security Number

Figure S2. Unadjusted Percentages of Cancer Survivors with ≥11 Months of Medicaid Enrollment Each Year Throughout 2015-2019 Who Received Any Cardiomyopathy Screening and Adhered to Screening Guidelines

Notes: ^a^ *P*=0.08 from the statistical (i.e., chi-square) test comparing the proportion with any cardiomyopathy screening by Medicaid expansion status of survivors’ residence.

^b^ *P*=0.05 from statistical (i.e., chi-square) test comparing the proportion that adhered to screening guidelines by Medicaid expansion status of survivors’ residence.

Table S1. Algorithm Used to Define Adherence Based on the Children’s Oncology Group (COG) Guidelines, Version 4 ^a^

| Age group | Cancer treatment exposure | Recommended frequency of echocardiogram (or equivalent imaging) ^b^ |
| --- | --- | --- |
| <1 year old | Any radiation + No anthracycline | Every 2 years |
|  | Any radiation + Any anthracycline | Every year |
|  | No radiation + <200 mg/m^2^ anthracycline | Every 2 years |
|  | No radiation + ≥200 mg/m^2^ anthracycline | Every year |
| 1-4 years old | Any radiation + No anthracycline | Every 2 years |
|  | Any radiation + Any anthracycline | Every year |
|  | No radiation + <100 mg/m^2^ anthracycline | Every 5 years |
|  | No radiation + 100-299 mg/m^2^ anthracycline | Every 2 years |
|  | No radiation + ≥300 mg/m^2^ anthracycline | Every year |
| ≥5 years old | Any radiation + No anthracycline | Every 5 years |
|  | Any radiation + <300 mg/m^2^ anthracycline | Every 2 years |
|  | Any radiation + ≥300 mg/m^2^ anthracycline | Every year |
|  | No radiation + <200 mg/m^2^ anthracycline | Every 5 years |
|  | No radiation + 200-299 mg/m^2^ anthracycline | Every 2 years |
|  | No radiation + ≥300 mg/m^2^ anthracycline | Every year |

Notes: ^a^ Our definition of adherence was based on the COG guidelines version 4.0, which were implemented in 2013 and thus align best with our study period.

^b^ For every year frequency, we required survivors to have at least one cardiomyopathy test claim in each year during 2015-2019. For every two-year frequency, we required survivors to fall into either of the two scenarios: (1) have at least one cardiomyopathy test claim in 2015, 2017, and 2019, respectively; or (2) have at least one cardiomyopathy test claim in 2016 and 2018, respectively. For every five-year frequency, we required survivors to have at least one cardiomyopathy test claim during the 5 years from 2015 to 2019.

Table S2. States by Medicaid expansion status as of 2019

| States that expanded Medicaid by 2014 (27) | Arizona, Arkansas, California, Colorado, Connecticut, Delaware, District of Columbia, Hawaii, Illinois, Iowa, Kentucky, Maryland, Massachusetts, Michigan, Minnesota, Nevada, New Hampshire, New Jersey, New Mexico, New York, North Dakota, Ohio, Oregon, Rhode Island, Vermont, Washington, and West Virginia |
| --- | --- |
| Late Expansion states (7) | Alaska, Indiana, Louisiana, Maine, Montana, Pennsylvania, and Virginia |
| Non-expansion states (17) | Alabama, Florida, Georgia, Idaho, Kansas, Mississippi, Missouri, Nebraska, North Carolina, Oklahoma, South Carolina, South Dakota, Tennessee, Texas, Utah, Wisconsin, and Wyoming |

Table S3. Factors Associated with Any Cardiomyopathy Screening and Adherence to Screening Guidelines among At-Risk Survivors with ≥11 Months Medicaid Enrollment Each Year

| Characteristics | N | Column % | Any Cardiomyopathy Test (yes vs. no) | | | Guideline Adherence (yes vs. no) | | |
| --- | --- | --- | --- | --- | --- | --- | --- | --- |
|  |  |  | Unadjusted % with Any Test  (row %) | Adjusted Probability  Difference  (i.e., Marginal Effects) | *P* | Unadjusted% Guideline Adherent  (row %) | Adjusted Probability Difference  (i.e., Marginal Effects)  (95% CI) | *P* |
|  | 887 | 100.0% |  | (95% CI) |  |  |  |  |
| *State policy-level predictor:* |  |  |  |  |  |  |  |  |
| ACA Medicaid expansion status |  |  |  |  |  |  |  |  |
| Non-expansion states | 230 | 25.9% | 22.6% | Ref. |  | 5.7% | Ref. |  |
| 2014 January expansion states | 513 | 57.8% | 29.8% | 8.97 (2.18, 15.77) | 0.01 | 10.9% | 5.90 (1.91, 9.89) | 0.004 |
| Late expansion states | 144 | 16.2% | 23.6% | 0.07 (-8.29, 8.43) | 0.99 | 11.8% | 5.97 (0.18, 11.76) | 0.04 |
| *ZIP Code-level predictor:* |  |  |  |  |  |  |  |  |
| Rurality of residence ^a^ |  |  |  |  |  |  |  |  |
| Small town or rural | 410 | 46.2% | 29.5% | Ref. |  | 10.5% | Ref. |  |
| Suburban | 272 | 30.7% | 25.4% | -4.52 (-11.79, 2.75) | 0.22 | 8.5% | -2.56 (-7.40, 2.28) | 0.30 |
| Urban | 205 | 23.1% | 23.9% | -6.04 (-13.81, 1.73) | 0.13 | 9.8% | -1.20 (-6.79, 4.40) | 0.68 |
| Neighborhood Distressed Communities Index ^b^ |  |  |  |  |  |  |  |  |
| 1 (prosperous) | 171 | 19.3% | 25.2% | Ref |  | 9.9% | Ref |  |
| 2 | 147 | 16.6% | 27.2% | 3.94 (-5.60, 13.48) | 0.42 | 10.2% | 0.03 (-6.98, 7.05) | 0.99 |
| 3 | 172 | 19.4% | 29.1% | 5.17 (-4.28, 14.61) | 0.28 | 9.3% | -1.14 (-7.65, 5.38) | 0.73 |
| 4 | 193 | 21.8% | 26.9% | 3.12 (-6.16, 12.41) | 0.51 | 11.9% | 1.31 (-5.90, 8.52) | 0.72 |
| 5 (distressed) | 204 | 23.0% | 26.5% | 3.00 (-6.72, 12.72) | 0.55 | 7.4% | -3.42 (-10.10, 3.26) | 0.32 |
| *Individual-level predictor:* |  |  |  |  |  |  |  |  |
| Age as of 2019 (years) |  |  |  |  |  |  |  |  |
| 27-39 ^c^ | 470 | 53.0% | 26.0% | -0.42 (-6.50, 5.66) | 0.89 | 8.5% | -2.70 (-6.81, 1.40) | 0.20 |
| 40-63.8 | 417 | 47.0% | 28.1% | Ref. |  | 11.0% | Ref. |  |
| Sex |  |  |  |  |  |  |  |  |
| Male | 377 | 42.5% | 26.8% | Ref. |  | 11.1% | Ref. |  |
| Female | 510 | 57.5% | 27.1% | -0.06 (-5.81, 5.69) | 0.98 | 8.6% | -2.72 (-6.78, 1.34) | 0.19 |
| Race and ethnicity |  |  |  |  |  |  |  |  |
| Non-Hispanic White | 647 | 72.9% | 27.7% | Ref. |  | 9.7% | Ref. |  |
| Non-Hispanic Black | 91 | 10.3% | 31.9% | 10.23 (-1.70, 22.15) | 0.09 | 11.0% | 5.62 (-3.91, 15.15) | 0.25 |
| Hispanic, other, or missing | 149 | 16.8% | 20.8% | -4.00 (-12.21, 4.21) | 0.34 | 8.7% | 0.76 (-5.05, 6.57) | 0.80 |
| Medicaid health plan type ^d^ |  |  |  |  |  |  |  |  |
| Fee for Service | 230 | 25.9% | 30.0% | Ref. |  | 10.0% | Ref. |  |
| Comprehensive managed care | 515 | 58.1% | 24.5% | -2.50 (-10.12, 5.12) | 0.52 | 8.4% | -0.23 (-5.29, 4.83) | 0.93 |
| Prepaid health plans | 108 | 12.2% | 28.7% | 1.84 (-8.54, 12.22) | 0.73 | 13.0% | 5.30 (-2.54, 13.13) | 0.19 |
| Behavioral health organization | 34 | 3.8% | 38.2% | 10.61 (-8.53, 29.75) | 0.28 | 17.7% | 7.84 (-5.24, 20.92) | 0.24 |
| Dual Medicare-Medicaid enrollment ^e^ |  |  |  |  |  |  |  |  |
| No dual enrollment | 437 | 49.3% | 24.3% | Ref. |  | 8.2% | Ref. |  |
| Any month of dual enrollment | 450 | 50.7% | 29.6% | 2.99 (-3.64, 9.61) | 0.38 | 11.1% | 1.41 (-3.19, 6.01) | 0.55 |
| Education attainment ^f^ |  |  |  |  |  |  |  |  |
| Some college or equivalent, college  graduate, or postgraduate | 390 | 44.0% | 28.2% | Ref. |  | 9.0% | Ref. |  |
| High school or lower, or unknown  education status | 497 | 56.0% | 26.0% | -1.45 (-7.73, 4.83) | 0.65 | 10.3% | 1.95 (-2.18, 6.07) | 0.36 |
| Presence of grade 3-4 non-cardiovascular medical conditions before 2015 |  |  |  |  |  |  |  |  |
| No | 439 | 49.5% | 29.5% | 3.36 (-2.53, 9.26) | 0.26 | 11.0% | 2.19 (-1.84, 6.21) | 0.29 |
| Yes | 448 | 50.5% | 24.5% | Ref. |  | 8.5% | Ref. |  |
| Presence of second cancer or recurrence of primary malignancy before 2019 |  |  |  |  |  |  |  |  |
| No | 714 | 80.5% | 25.5% | Ref. |  | 9.1% | Ref. |  |
| Yes | 173 | 19.5% | 33.0% | 5.70 (-1.95, 13.36) | 0.14 | 12.1% | 1.77 (-3.37, 6.92) | 0.50 |

Notes: Abbreviation: CI = confidence interval. Ref. = reference.

At-risk: those who received anthracycline chemotherapy, radiation to chest or heart, total body irradiation, or a combination of these treatments.

^a^ This measure was based on the 2010 U.S. Census urban and rural classification and urban area criteria to classify a ZIP code as urban, suburban, small town, or rural.

^b^ Distressed Communities Index (DCI) was calculated based on seven neighborhood-level measures: no high school diploma, housing vacancy rates, adults not working, poverty rate, median income ratio, changes in employment, and changes in establishments. The DCI was then classified into quintiles: prosperous (quintile 1), comfortable, mid-tier, at risk, and distressed (quintile 5). The DCI used in this analysis was built from the U.S. Census Bureau’s American Community Survey 5-year estimates covering 2014-2018 and the Census Bureau’s Business Patterns datasets for the same years.

^c^ The minimum age (as of 2019) of our analytic sample was 27 years old.

^d^ Medicaid plan type was measured in 2019.

^e^ Dual Medicare-Medicaid enrollment was defined as at least one month enrolled in Medicare and Medicaid over the 5-year study period (2015-2019).

^f^ The measure of education was from the most recent CCSS survey data within our study period; if this information was unavailable, we used data from the preceding survey that was most recent.
